# Combining soft robotics and telerehabilitation for improving motor function after stroke

**DOI:** 10.1017/wtc.2023.26

**Published:** 2024-01-26

**Authors:** Tommaso Proietti, Kristin Nuckols, Jesse Grupper, Diogo Schwerz de Lucena, Bianca Inirio, Kelley Porazinski, Diana Wagner, Tazzy Cole, Christina Glover, Sarah Mendelowitz, Maxwell Herman, Joan Breen, David Lin, Conor Walsh

**Affiliations:** 1 John A. Paulson School of Engineering and Applied Sciences, Harvard University, Cambridge, MA, USA; 2 Whittier Rehabilitation Hospital, Bradford, MA, USA; 3 Center for Neurotechnology and Neurorecovery, Department of Neurology, Massachusetts General Hospital, Boston, MA, USA; 4 VA RR&D Center for Neurorestoration and Neurotechnology, Rehabilitation R&D Service, Department of VA Medical Center, Providence, RI, USA

**Keywords:** soft robotics, stroke rehabilitation, telerehabilitation, wearable robotics

## Abstract

Telerehabilitation and robotics, either traditional rigid or soft, have been extensively studied and used to improve hand functionality after a stroke. However, a limited number of devices combined these two technologies to such a level of maturity that was possible to use them at the patients’ home, unsupervised. Here we present a novel investigation that demonstrates the feasibility of a system that integrates a soft inflatable robotic glove, a cloud-connected software interface, and a telerehabilitation therapy. Ten chronic moderate-to-severe stroke survivors independently used the system at their home for 4 weeks, following a software-led therapy and being in touch with occupational therapists. Data from the therapy, including automatic assessments by the robot, were available to the occupational therapists in real-time, thanks to the cloud-connected capability of the system. The participants used the system intensively (about five times more movements per session than the standard care) for a total of more than 8 hr of therapy on average. We were able to observe improvements in standard clinical metrics (FMA +3.9 ± 4.0, *p* < .05, COPM-P + 2.5 ± 1.3, *p* < .05, COPM-S + 2.6 ± 1.9, *p* < .05, MAL-AOU +6.6 ± 6.5, *p* < .05) and range of motion (+88%) at the end of the intervention. Despite being small, these improvements sustained at follow-up, 2 weeks after the end of the therapy. These promising results pave the way toward further investigation for the deployment of combined soft robotic/telerehabilitive systems at-home for autonomous usage for stroke rehabilitation.

## 1. Introduction

Every year, in the United States alone about 800 k people have a stroke (CDC, 2022). Thanks to advances in medical technologies, more people are surviving this event, however living with the subsequent chronic impairments and disabilities associated with this condition (Heart.org (n.d.)). Given that 60–90% of individuals post-stroke have lasting arm impairments (Kwakkel, 2003; Dobkin, 2005), the majority of stroke survivors face challenges in performing activities of daily living (ADLs), with negative consequences on independence and quality of life. Hand function is critical for functional recovery after stroke (Takahashi et al., 2008; Bae et al., 2015; Hsieh et al., 2018; Lee and Shin, 2021) and directly correlates with recovery of performance of ADLs (Bland et al., 2008; Kim, 2016).

Rehabilitation focused on high-intensity, repetitive task practice has been shown to improve hand function (Bütefisch, 1995; Ward, 2019). However, programs to achieve this tend to be labor intensive and expensive and thus they are not widely available in the majority of rehabilitation settings (Duret et al., 2019). The current standard of care is known to be performed at low intensity (Lang et al., 2009), orders of magnitude below what is needed to drive optimal recovery (Jeffers et al., 2018). In addition, the amount of standard care therapy or dosage is also limited both for inpatient and for outpatient rehabilitation (Winstein, 2016), with the latter not always covered by insurance (Insurance Appeals, 2022). There is thus an ongoing need for improved therapy tools to increase both intensity (i.e., amount of repetitions per therapy session) and dosage (i.e., duration and frequency of therapy sessions) significantly (Coscia et al., 2019).

A number of technologies and tools have been developed to try to address these issues. These include telerehabilitation (Richmond et al., 2017), spring-based passive systems (Adams et al., 2019), sensorized gloves for quantitative hand movement measurement and feedback (Sanders et al., 2020), functional electrical stimulation (FES) (Franck et al., 2018), virtual reality and gaming (Qiu et al., 2020), and robotics (Proietti et al., 2022). All these approaches have shown promise, for example, telerehabilitation increased therapy hours out-of-clinic (Laver et al., 2020), and achieved the same benefits of dosage- and intensity-matched in-person therapy (Cramer et al., 2019). However, telerehabilitation, passive and sensorized gloves, virtual reality, and gaming have been primarily explored with individuals who possess enough residual upper limb function to complete exercises, especially if at home without hands-on support from a therapist (Piron et al., 2009; Wittmann et al., 2016; Dodakian et al., 2017; Cramer et al., 2019; Qiu et al., 2020; Sanders et al., 2020; Thielbar et al., 2020). This is a clear limitation given that 50% of stroke survivors are chronically disabled with severe impairment, spasticity, and hand tightness (Donkor, 2018). FES was indicated as an effective treatment when targeting proximal joints, for example, for shoulder subluxation (Vafadar et al., 2015), but evidence of the advantage of its application to the hand is still missing (Coscia et al., 2019). Robotics, on the other hand, has the potential of being able to assist stroke survivors at any level of impairment and a few hand robotic *exoskeletons* have reached the market and have undergone preliminary clinical trials (e.g., Kutner, 2010; Sale, 2012; Kim et al., 2019; Milia et al., 2019). However, traditional robots can be challenging to align with the ultra-compact multi-degree of freedom (DOF) human hand, are complex to use, and are generally expensive, thus limiting the adoption out of high-tech clinics and the usage at-home unsupervised (Langan et al., 2018). Soft robotic gloves, made of textiles and using cables (e.g., Xiloyannis et al., 2016; Ghassemi et al., 2018; Kang et al., 2019; Yurkewich et al., 2020), pneumatics (e.g., Coffey et al., 2014; Polygerinos et al., 2015; Zhou et al., 2019; Correia et al., 2020; Lai et al., 2023a, 2023b; Lim et al., 2023), or serial elastic actuators (e.g., Xu et al., 2023) as the actuation mechanism, have been presented more recently as an alternative to traditional exoskeletons (Chu and Patterson, 2018; Proulx et al., 2020; Akbari et al., 2021). They potentially allow reduced weight, lower form factor, and increased portability, improved capability to interact with real-life items during ADLs, suitability for safe and independent use at home, and generally lower costs than exoskeletons. However, exciting developments in device hardware have not yet come with extended evaluation on stroke survivors, in particular outside of a lab setting, limiting the demonstration of their effectiveness.

A recent emerging research effort aims at combining the advantages of rehabilitation robotics with the benefits of telerehabilitation, using a robot to achieve high-intensity and high-dosage rehabilitation at a patient’s home. The intervention can be fully or partially remotely supervised by a therapist, who can provide guidance, assess progress, and help the patient in adhering to the therapy program. To date, only a small number of home-based studies have been performed using rehabilitation robots aimed at improving hand function, using either soft (Bernocchi et al., 2018; Radder et al., 2019) or rigid (Wolf et al., 2015) robotic devices. Nijenhuis et al. (2015, 2016) tested a rehabilitation system at home consisting of a passive commercial exoskeleton for the arm in combination with a prototypal passive exoskeleton for the hand, actuated with springs and elastic tension cords. Apart from the initial setup of the device with the participants, none of these studies required any hands-on support by a therapist. However in Nijenhuis et al. (2015, 2016) and Bernocchi et al. (2018) a caregiver was needed to wear the device on the impaired hand. The therapist always only interacted remotely with the participants to help in adhering to the program. By either applying passive mobilization to the hand (Bernocchi et al., 2018) or active assistance in games (Nijenhuis et al., 2015, 2016; Wolf et al., 2015; Radder et al., 2019) or ADLs (Radder et al., 2019), they were able to show some improvements in common clinical metrics (e.g., FMA-UE, BBT, ARAT, JTHFT). Noticeably, these studies enrolled stroke individuals with mild impairments (Nijenhuis et al., 2016; Radder et al., 2019) or those in the early stages of recovery, that is, sub-acute (Wolf et al., 2015; Bernocchi et al., 2018), in which spontaneous recovery is still possible.

**Figure 1.**
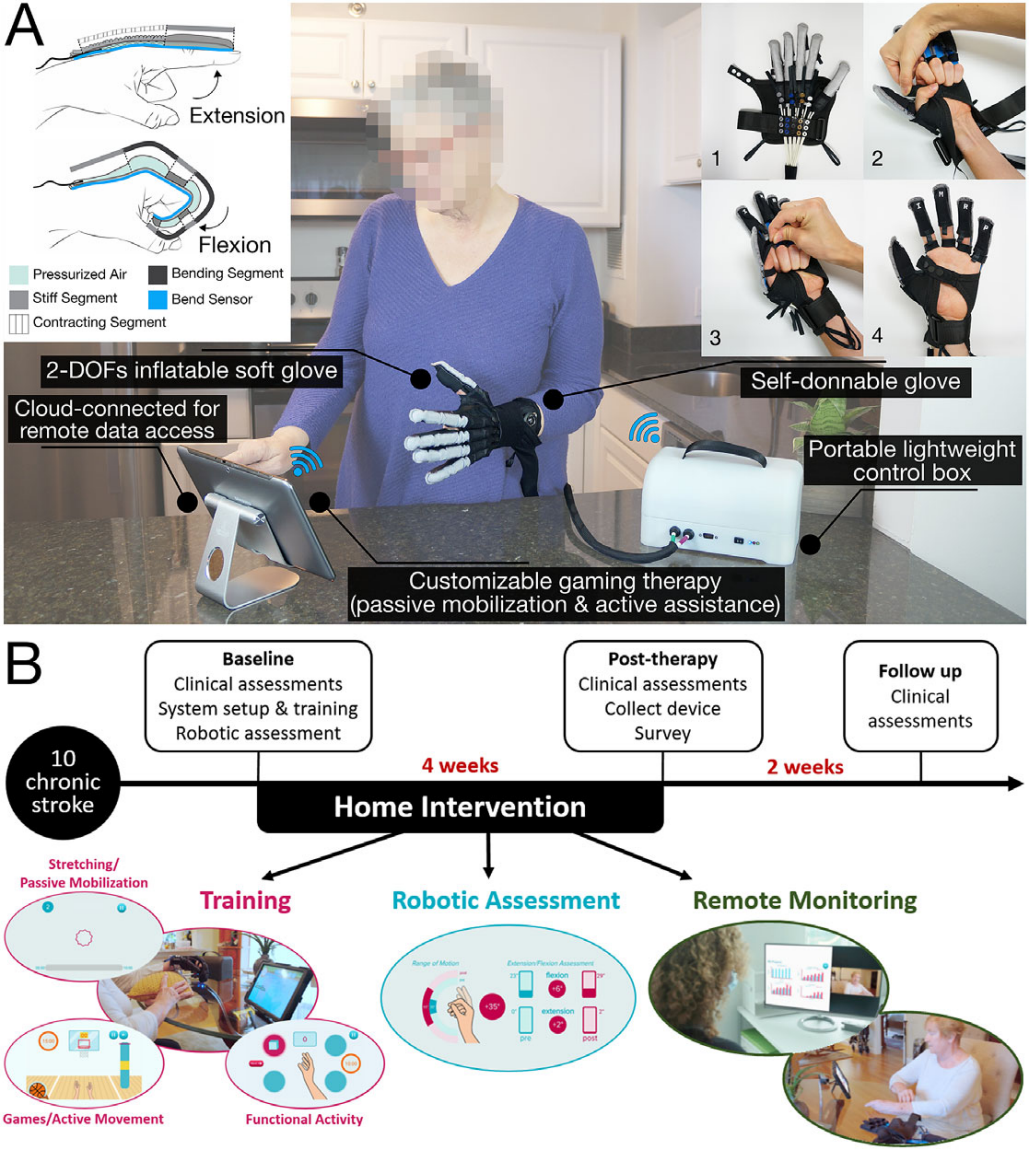
(a) Soft robotic glove for post-stroke rehabilitation at home. A self-donnable textile-based inflatable glove controls finger movement along 2 DOF, extension or flexion. A bend sensor measures the angle of rotation at the tip of the index finger with respect to the palm of the hand. The fully unsupervised therapy is led by a custom software interface, guiding the patient through the sessions with passive and active exercises. Raw and processed data are stored in a connected database in real-time, accessible by the study clinical team. (b) Pilot study protocol overview. Ten chronic stroke survivors (baseline FMA = 33.8 ± 9.3, Table 1) were enrolled in a 4-week intervention performed at home. Baseline clinical assessments included FMA-UE (primary outcome), MAS, GWMFT-FAS, GWMFT-TIME, BBT, MAL, COPM, and grip strength (secondary outcomes). The home intervention consisted of three parts: app-based training, automatic assessment measured by the robot, and remote monitoring by an occupational therapist who did not participate in any training session. Robotic outcomes were measured inter- and intra-session to track progress overtime. At the end of the intervention, the device was collected and the same baseline clinical measurements were performed post-therapy. A system usability scale was also collected to determine user satisfaction. A 2-week follow-up assessment concluded the study.

In this article, we present the design and a preliminary evaluation of the feasibility of a cloud-connected soft robotic telerehabilitation system for hand rehabilitation after stroke that combines active assistance, user guidance and engagement, and quantitative assessment, see Figure 1.A. The system is the result of a user-centric design process with multiple iterative design cycles guided by deep engagement with patients and clinical stakeholders. Key aspects of the design include real-time and post-session visualization of both usage and performance metrics for the stroke survivor, remote access to data and metrics by a therapist, a self-donnable glove design, a user intention-detection strategy for participant active interaction with an intuitive software interface that includes a number of games. Importantly, the presented system is suitable for individuals at all levels of impairment, including those in the chronic stage of recovery with moderate to severe impairments. The key innovation of this investigation lays in the maturity of the technological development of the device and of the integration of all the components. This allowed the use of the robot at the patients’ home, unsupervised, with infrequent check-ins with therapists such that stroke survivors were in-charge of their therapy experience. Here, we present the evaluation of this technology in a 1-month home-based pilot intervention with 10 chronic stroke survivors where we evaluated changes in clinical outcomes and overall system usability (Figure 1.B). The purpose was not to conduct an extensive clinical trial, with comparison to a control group, but rather to assess the feasibility of this approach through a pilot study with a limited number of selected participants.

## 2. Methods

### 2.1. User-centric design

The robotic platform presented in this work is the result of a user-centric design process, consisting of several iterations with end-users (stroke survivors, clinicians, and experts in the field of rehabilitation). This effort led to the final design of both the robotic glove and the custom software interface from the end-user perspective. In particular, as part of the system development process, a total of nine interactive meetings or *focus groups* (six with stroke individuals, three with therapists), and an online survey were conducted.

Six focus groups involved a total of seven individuals post-stroke (each participant joined multiple focus groups, each session had 2–5 participants), two of whom were later enrolled in the study protocol. Feedback was requested on two main points: (i) describing the participant’s current rehabilitation strategies and (ii) their reaction to different iterations of component and system prototypes (both appearance-related and functional features of the system). For the glove, comfort, esthetic, and ease-of-use were discussed, as well as what features would enable independent use in the home. For the software, the focus was on ease-of-use, user engagement (including data participants wanted to review at the end of every session and track during the intervention), and three different game prototypes tested by the focus group participants.

Three additional focus groups involved 11 occupational therapists (OTs) and physicians from a range of backgrounds (academia, home care, outpatient therapy, and rehabilitation hospitals). A large number of design iterations and preliminary tests on healthy individuals were shown to these specialists to garner design feedback. Thematic analysis (Braun and Clarke, 2006) was used to break down and organize key categories to address in the following design iteration. Themes identified during the analysis included user motivation (both gaming and data feedback/session reports), independent use, and device specifics.

The online survey was completed by 24 OTs and physical therapists (PTs) and was composed of two parts consisting of a total of 75 questions (24 questions between ratings 1–10 and rationale behind the score, 51 open-ended questions). The first part of the survey focused on accessing current clinical practice, while the second part on collecting feedback on the concept of at-home technology for rehabilitation. In particular, respondents were asked to comment on current clinical strategy applied by therapists and main challenges to recovery, goal of home-based therapy and proposed scheduling, if any technology was already available and commonly used, ideas for new approaches or technology, the importance of standard assessment and how to track progress, the role of motivation and approaches to boost up engagement by patients.

Finally, an initial version of the glove robot was also demoed in an expo booth at the 2019 American Congress of Rehabilitation Medicine (ACRM) Rehabilitation Conference where additional opinions were collected by experts of the field.

### 2.2. System design

The robot consists of two main components: (i) a soft robotic textile-based glove with a portable pneumatic control box, and (ii) a custom software interface running on an off-the-shelf tablet (Galaxy Tab A, Samsung, South Korea). The glove and the control box are connected through pneumatic lines to control extension and flexion of the user’s hand, and a data line for the information from the glove-integrated sensor. The tablet instead connects to the control box over Bluetooth and to the cloud over Wi-Fi to automatically back up session data. The control box and thus the glove are wall-powered.

#### 2.2.1. Soft robotic glove and control box

A detailed description of the textile-based glove and its fabrication process can be found in previous works (Zhou et al., 2019; Correia et al., 2020). In brief, the glove consists of a hand wrap with five-finger actuators. Each actuator has two chambers, one for extension and one for flexion, separated by textile material. For extension, a first thermoplastic polyurethane (TPU) bladder is inserted into a stiffer textile woven tube that radially contracts or expands based on the bladder’s pressure. For flexion, a second TPU bladder is inserted into a more compliant textile woven tube that bends upon inflation around the finger joints. Both bladders have a heat-sealed tube to connect to the control box.

A few additional features were added to this version of the glove, aimed at improving self-donnability. The new glove design can be donned with one well-functioning hand, thus aimed at independent use by stroke survivors. Users don the glove with the control box stationary on a table. The hand wrap has an open palm with a thumb slot and a wrist strap. This design allows for users to place their hand into the device even if they are significantly impaired on their paretic side. On the back of the wrap, there are corresponding snaps and a fabric tube for each finger actuator to be properly positioned on the glove. The snaps are color-coded to simplify donning. Additionally, a boning attachment could be added to the thumb for optimal thumb alignment during object pick-up, especially for tasks involving a pinch grip.

In addition to these features, a bend sensor (*2-Axis Soft Flex Sensor*, Bendlabs, Farmington, UT) was also integrated in the glove. The sensor is attached to the side of the index finger with rubber silicone adhesive (Sil-Poxy, Smooth-On, Inc., East Texas, PA) to gather angle measurement information. The sensor is used for remote assessments and to estimate intention detection for movement during the games. In particular, the intention detection algorithm uses data from the sensor to decide when the user is attempting to open or close their hand. To make the detection, the algorithm uses two low-pass filters, one at 5 Hz and the other at 1 Hz. The 5 Hz filter is responsible for removing the noise from the sensor, while still able to follow most hand movements from the user. The 1 Hz filter is responsible for keeping track of a baseline hand position, for example when users slowly move from one hand posture to another. When the difference between the output of these two filters is higher than a threshold, an intention detection is triggered and the glove is pressurized (either in flexion or extension) to a set value.

The hardware controlling the glove and interfacing with the software is within a 3D-printed custom control box. The box is lightweight (2.27 kg) and compact (24 cm × 16 cm × 14.5 cm). The whole system is Federal Communications Commission (FCC) tested and approved. Within the control box, a custom steel plate separates the electronic components from the pneumatic components. On the electronic side, a microcontroller (BeagleBone Black Wireless, Beaglebone, Rochester, MI) with a custom printed circuit board allows for on/off control of high current actuation for pumps and solenoid valves, sensor reading, and communication between the control box and tablet. Electronic safety features are also included (e.g., flyback diodes, electrostatic discharges protection and reverse polarity protection).

The pneumatic system utilizes two pumps (ROB-10398, Sparkfun Electronics, Niwot, CO) in parallel. These pumps connect to a series of three-way, 2 position, normally closed valves (AVS-3211-24D, NITRA Pneumatic, Port Sanilac, MI) that lead to an extension and flexion outlet. Each manifold chamber connects to a pressure sensor (SSCDANN100PAAA5, Honeywell, Charlotte, NC, USA) so that the glove pressure in extension or flexion can always be monitored and regulated. Before the outlet connection to the flexion and extension tubing, there is a normally open valve (BVS-32A1-24D, NITRA Pneumatic, Port Sanilac, MI) that vents the system in case of power loss.

Outside the box are the sensor Lemo (EGG.00.304.CLL, LEMO, Rohnert Park, CA) connection, and pneumatic flexion and extension quick connects. The quick connects are color-coded with the pneumatic lines from the glove to reduce plugging errors. A single LED-lit button was used for powering the device as well as acting as the emergency stop.

#### 2.2.2. Software interface

The software interface walks participants through a self-administrated session of therapy. A few initial settings are needed to be input by the user in the software interface (e.g., paretic side, skin tone, gaming difficulty, and selecting sensor vs. non-sensor mode). Participants cannot change the settings without the consent of the OT or research team. Before starting a session, participants go through a safety checklist to ensure that they are properly prepared for the session. The checklist includes clearing the table of liquids, correctly setting up the control box, donning the glove, and securing cables out of the way. Once a participant goes through their safety check, the workflow of each session consists of assessments, stretching, and functional activities. During each session, the tablet was connected to the cloud to automatically back up user data. The recorded information were: assessment metrics, like range of motion (ROM) and flexion/extension force pre- and post-session, time and repetitions for each section as well as for the total session and amount of active time spent during the session. Data was stored automatically after the completion of each session and could be accessed by both the study team and the OT assisting the participant at any time.

### 2.3. Participant population

Ten chronic stroke survivors were recruited to participate in a 4-week at-home hand rehabilitation study. Participants were recruited from rehabilitation centers in the greater Boston area and gave their written informed consent before taking part in the study, approved by the Harvard Medical School Institutional Review Board (IRB13-3418). The inclusion criteria for this study were: (i) being 18–85 years old; (ii) being fluent in English; (iii) having suffered a stroke more than 6 months ago; (iv) absent of COVID-19 symptoms or diagnosis within 2 weeks of initiating study and throughout study; (v) maintains a COVID-19 safe home and agreeable to researchers coming to their home to perform clinical assessments at initial, final, and follow-up visits with precautions in place (masks, HEPA filter, temperature and symptom screening). Participants were excluded if (i) the scored less than 23 on a mini-mental state exam (MMSE) (Folstein et al., 1975); (ii) they had open wounds on the impaired limb; (iii) they had any pain when ranging the impaired limb; (iv) they scored greater than 3 on the MAS; and (v) they were receiving additional upper-limb focused therapy while part of the study. No participants received Botox during their participation. Please refer to Table 1 for a detailed look at the patient population in this study.

**Table 1.** Participants data

		Time post	FMA-UE		Stroke	
	Age	stroke (m)	baseline	Sex	Type	Side
P1	63	60	21	M	I	R
P2	47	66	27	M	H	L
P3	62	113	36	M	H	L
P4	63	60	24	M	I	L
P5	60	50	39	F	H	R
P6	62	12	24	M	H	R
P7	76	24	51	F	I	L
P8	63	31	42	F	H	L
P9	38	12	42	F	I	R
P10	51	11	32	F	I	L
Avg	58.5	43.9	33.8	5F/5M	5H/5I	6 L/4R
SD	10.0	30.8	9.3			

*Note.* Ten chronic stroke (~4 years after stroke event on average) individuals were enrolled in the study. Half of the participants were severe-to-moderate impaired (FMA-UE ≤ 32), half mild impaired.

### 2.4. Study design

Participants began their 4-week regimen with a baseline examination in their place of residence which included several standard clinical assessments: FMA-UE (primary outcome), MAS, GWMFT, BBT, MAL, and COPM (secondary outcomes). Grip strength was also measured using a digital hand dynamometer (*EH101*, Gripx, Anderson, SC). The choice of FMA-UE as the primary outcome was driven by the severity of impairment in our testing population, which rendered more complex upper limb assessments, like the ARAT or the 9 Hole Peg Test, involving various grasping and reaching prompts, inappropriate. All baseline evaluations were performed by one of the three OT members of the research team, who therefore were not blinded to the protocol, and are available in Supplementary Table S1.

A full system was prepared by the research team, including an appropriately sized and sided glove for each participant. The OT introduced the device to each participant, which included choosing an optimal location in their house for using the device as well as instructions on each training phase. In addition to the robot, supplementary equipment was also provided. A foam block was provided to perform simulated functional activities guided by the app. If recommended by the OT, a foam armrest was also used to prop up the paretic hand to maintain a usable position. Finally, to avoid pausing glove use mid-trial in case of any hardware issues, participants were given a back-up glove.

Participants were encouraged to partake in a minimum of three full sessions per week as part of the study, but free to complete as many sessions as they were able. The duration of the single session was also self-selected, despite a suggestion of a minimum of 45 total minutes (including the setup of the robot, donning, and doffing). During the 4-week of intervention, virtual remote monitoring by an OT was provided to troubleshoot, train, and encourage habitual use of the device as well as guide the participant toward active attempts of affected upper limb engagement in self-selected daily life activities identified in the COPM. The OT intervention was designed to be decreasing in frequency throughout the month was to promote self-initiated engagement by the participants such that they became more and more in charge of the intervention and therapy experience. Moreover, we wanted to test if this approach changed the use of the glove, in terms of the number of sessions, intensity, and duration of each session. Virtual monitoring started with four sessions per week and reduced to two sessions per week, with an average of 15–30 min per session.

In-person evaluation of the abovementioned standard assessments was performed again at the end of the 4-week intervention. At this point, the device was also returned. Participants returned to their daily lives without the device and a 2-week follow-up (in-person) was performed to measure improvement over time. A System Usability Survey (SUS) was also administered at the end of the 4-week intervention.

Each training session consisted of several assessments and activities for the participants to go through on the software interface in sequential order. First, participants underwent a maximum ROM assessment followed by a flexion and extension force assessment to establish a baseline hand performance at the start of each session. A total of 2.5–5 min of passive flexion and extension stretching cycles of the hand by the robot followed, depending on the session length. Participants could then choose between three different games that incorporated hand movements as the active controller for 10–20 min (again depending on the session duration). Next, a functional activity (5–10 min) provided a way for participants to further develop neural pathways to control their paretic hand to grasp and release objects using a timer. The same two assessments (ROM, force) were performed a second time toward the end of the session. Participants were then given the opportunity to go through a second round of stretching, of the same duration of the first one. At the end of each session, a final report was provided to show the performance of the participant across the session. The report showed the number of repetitions completed in the session and across the studies duration as well as any improvements during the session in either assessment.

### 2.5. In-app/robotic assessments

#### 2.5.1. Maximum range of motion assessment (RA-ROM)

The user was asked to actively open and close their hand with the glove deflated. The total angular displacement of the finger was the user’s active range of motion. A simple user interface guided users through opening and closing their hand while automatically measuring maximum angular displacement with the bend sensor during the opening and closing of the hand. It is important to notice that, given the unsupervised condition of the experiment and the nature of the sensor, we did not measure absolute angles of finger flexion/extension but only maximum angular displacement with respect to a starting hand pose.

#### 2.5.2. Flexion and extension strength assessment (RA-STR)

The user was asked to close or open their hand as strongly as possible against the actuators inflated. Specifically, to measure finger extension strength, the user tries to push the fingers into flexion while being held in extension by the inflated actuators. To measure flexion strength, the user pushes the fingers into extension while being held in flexion by the inflated actuators. By measuring the ROM generated by the user at a given actuator pressure, we were able to track strength increase/decrease over time. In theory, a calibrated model mapping ROM and actuators inflation would allow to estimate exerted force as well, but this was out of scope of this investigation, and only trends of estimated strength were considered.

### 2.6. Statistical analysis

Nonparametric methods were applied due to the small sample size (*n* = 10). We used a Wilcoxon matched pair signed-rank test to assess the statistical difference of the absolute variables between time points (e.g., baseline vs. 4-week of intervention). The significance level is reported when exceeding standard *p* values of significance (*p* < .05, marked with single asterisk *). The analysis was performed using Matlab 2022a (Mathworks, Natick, MA).

## 3. Results

### 3.1. User-centric design process

**Figure 2.**
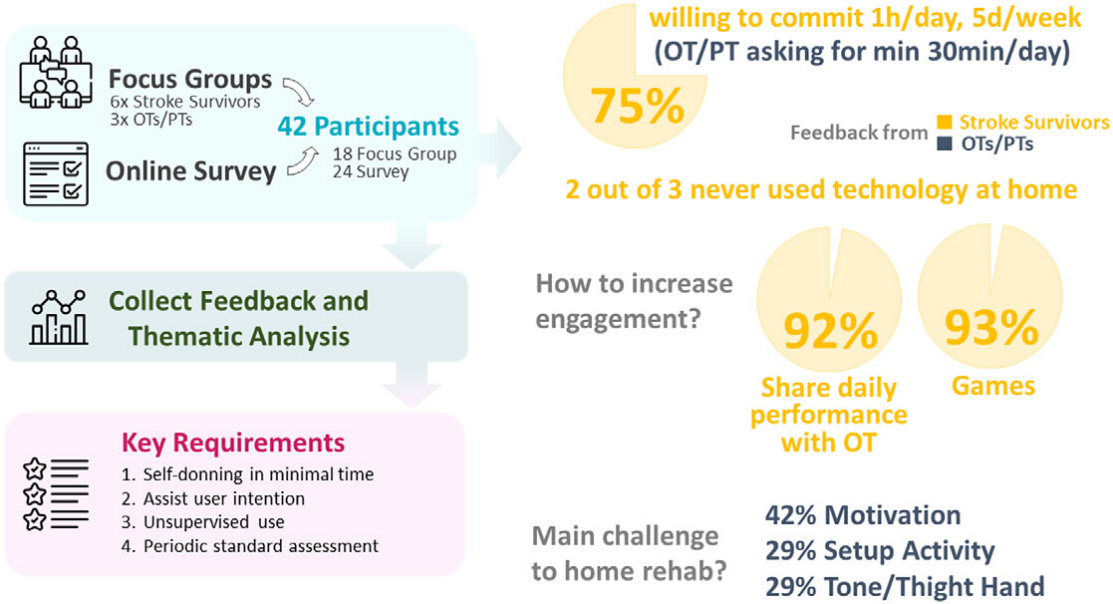
User-centric design process and key results. Forty-two participants among stroke survivors, occupational and physical therapists, and physical therapists provided feedback through either focus groups or an online survey. These pre-study data guided the design of the combined soft robotics-telerehabilitation approach. In yellow, feedback from stroke survivors and in blue is from OTs/PTs.

An overview of the user-centric design process is illustrated in Figure 2. While all participants (PT, OT, and stroke individuals) looked for a technology with unsupervised home-rehabilitation capabilities, interestingly, a large degree of variability was observed for the stroke survivors willingness to commit to a home-based rehabilitation robotics protocol. The majority (75% of participants) said they would be free for close to an hour at least 5 days a week, while the remainder were equally split between those available for as much time as needed for recovery, and those willing to spend at least 30 min on recovery, 3 days a week. The feedback from the OTs and PTs highlighted the importance of completing home rehabilitation alone, with exercises every day for at least 30 min/day. When asked what might lead to increased engagement in a program, 92% of the stroke survivors highlighted an interest in sharing their daily performance with their OT in some way and 93% said that games would make their rehabilitation experience more enjoyable. This was in line with OT/PT answers to the survey, while motivation (42%), need of assistance to setup activities (29%), and tone/tight hand (29%) were described as the main typical challenges in completing home-based rehab. From both stroke survivors and OTs there was a strong consensus that the standard of care is “boring.” Key system requirements that emerged were: (i) ability to self-don the glove in minimal time, (ii) assistance should be triggered by the participant for at least part of the activities, (iii) unsupervised use, thus not requiring the presence of a therapist or a caregiver, and (iv) importance of receiving periodic standardized assessment to track recovery. All interviewed were open to and comfortable with the concept of telerehabilitation and using remote virtual calls, despite one-third of the interviewed OT/PT never used any advanced technology with their clients and two-third of their clients never used any technology at home.

### 3.2. Positive usability and self-donning experience

The 10 chronic stroke participants were given one therapist-led in-person training at the start of the intervention and following that, were able to achieve successful independent usage of the device. Despite 7 of the 10 participants living alone and having no caregiver present for troubleshooting or physical assistance, only one reported needing occasional assistance to don the device, and all the other nine participants were always able to don and doff the device alone and use the system completely independently. Our system usability survey confirmed a positive experience with a system usability scale (SUS) outcome of 77.2 ± 14.7, a value indicating high probability for acceptance in the field (Bangor et al., 2009).

### 3.3. Intensive therapy performed at any level of impairment

**Figure 3.**
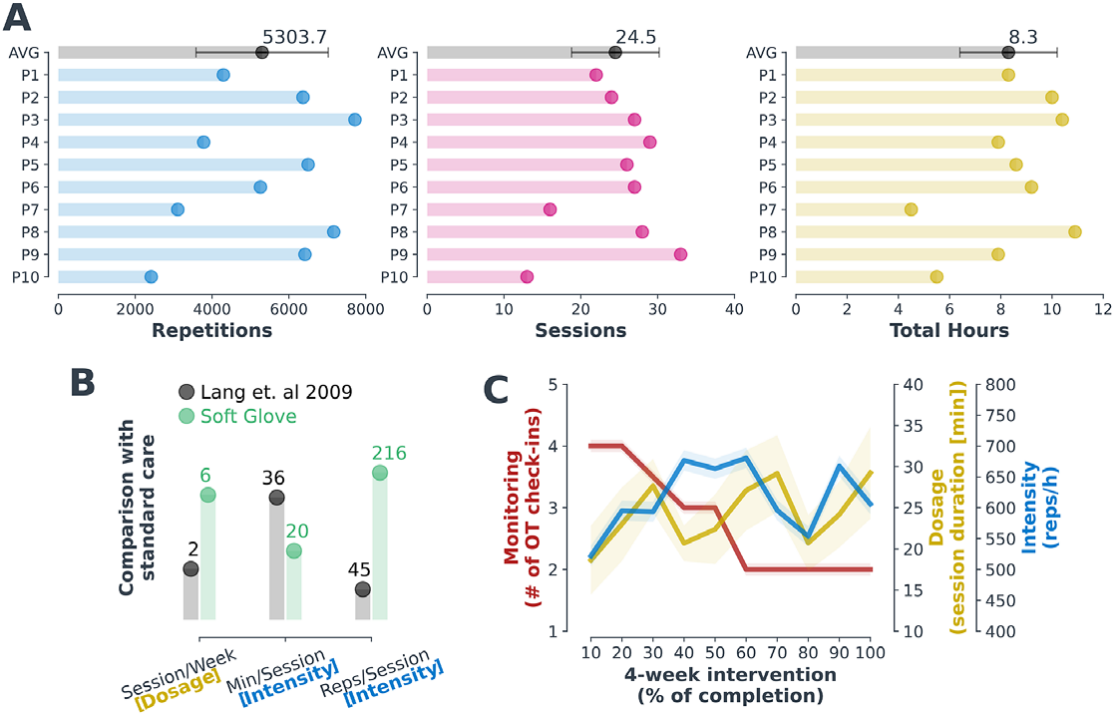
(a) Intensity (number of repetitions) and dosage (number of sessions and total therapy hours) of the 4-week robot-assisted therapy. On average, each participant performed more than 5000 repetitions in 25 sessions, for a total of 8.3 hr of exercise. A repetition is defined as an opening or closing of the hand from a rest position. Duration of practice is the effective use of the robot to perform movements, thus excluding setup procedures. (b) Soft glove intervention versus standard care. Average data from this study and from standard care literature data (Lang et al., 2009). The unsupervised robotic intervention allowed for higher dosage (sessions per week) and more intense therapy (repetitions per session) in a shorter session duration. (c) Therapy dosage and intensity versus OT monitoring. Participants engagement and activity did not decrease, despite the reduction of the OT monitoring time as by protocol design. This demonstrates the efficacy of the technology and the positive experience for the participants. It is important to underline that the OT check-in never consisted of monitoring a training session but focused on helping the patient to maintain adherence to the program. Values represent average and standard error over the whole population.

The participants used the system consistently and at high intensity over the course of the study as shown in Figure 3a. On average, with the system, participants performed a total of over 5,300 movements (i.e., hand opening or closing) in 25 sessions, thus more than 8 hr of combined passive and active exercise during the 4 weeks of intervention (20 ± 5 days on average with at least one session). On average for each session, they completed 216 movements; remarkably, this rate is an order of magnitude higher than what is typically experienced by stroke individuals during standard care (Lang et al., 2009), see Figure 3b. Noticeably, despite OT virtual monitoring reducing over the course of the intervention as by design (average of 15–30 min per monitoring session; 4 sessions the first week, and only 2 the last week), both intensity and dosage of the therapy were sustained during the 4 weeks of availability of the robot at home as shown in Figure 3c.

### 3.4. FMA-UE improved over the MCID

**Figure 4.**
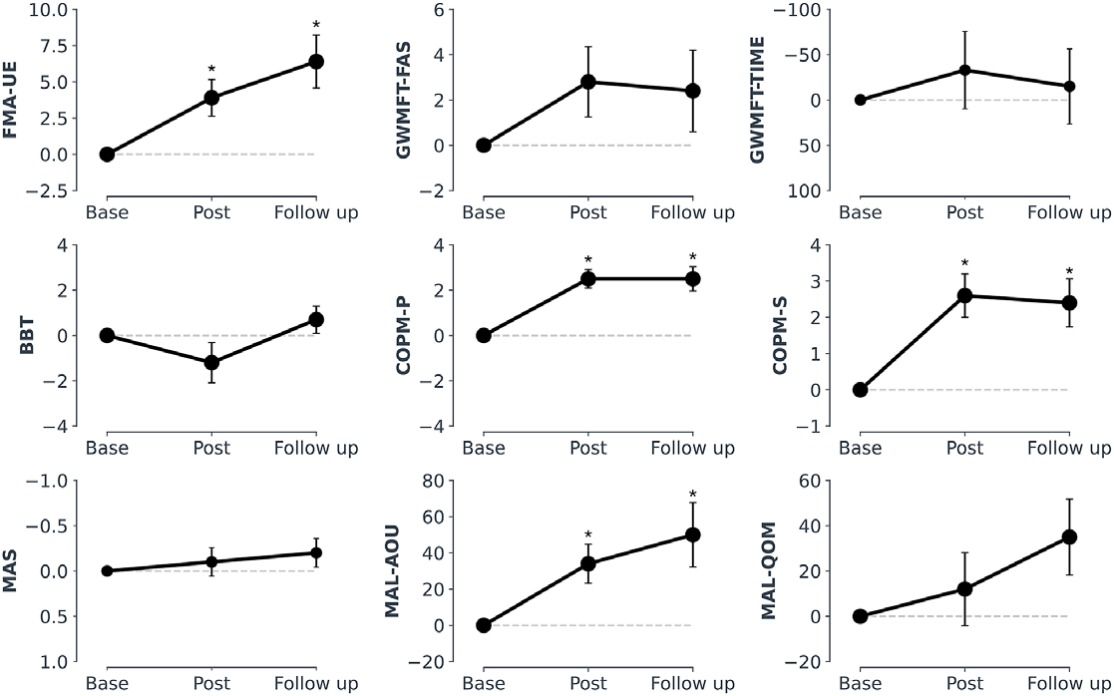
Standard clinical metrics results: gains over the baseline. Average data and standard error with respect to the baseline values. Improvements are represented by upward trends (GWMFT-TIME and MAS *y*-axes are inverted). FMA-UE, COPM, MAL-AOU significantly improved and scored above the MCID. All values are unit values of the specific metric (e.g., BBT is in number of blocks per minute) but GWMFT and MAL, reported as a percentage. Asterisk indicates the statistical significance of the absolute values with respect to the baseline after Wilcoxon signed-rank test (*p* < .05). Please refer to Supplementary Table S1 for all absolute values.

Changes in FMA-UE, one of the most used metrics for assessing motor recovery after stroke and the primary outcome of this study, are illustrated in Figure 4. We observed an average of 3.9 ± 4.0 points improvement at the end of the intervention and 6.4 ± 5.8 points at the follow-up. The associated improvements in terms of absolute values are statistically significant with respect to the baseline values (after a Wilcoxon signed-rank test, *p* < .05). Moreover, at follow-up, they are larger than the minimal clinically important difference (MCID, 5.25 points in chronic stroke individuals; Page et al., 2012).

**Figure 5.**
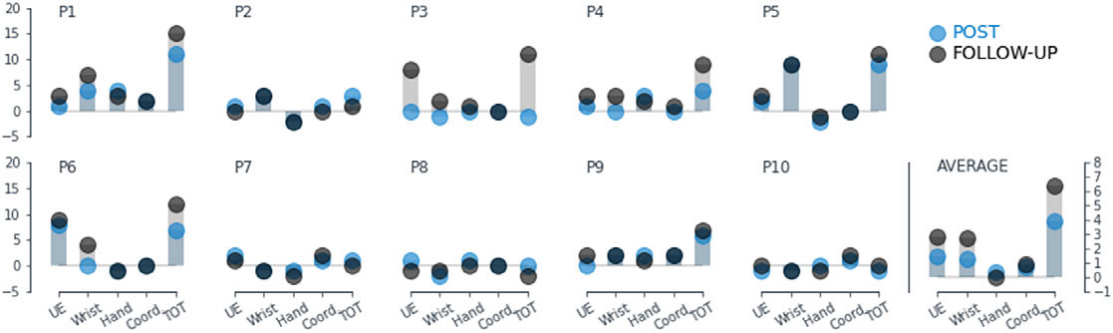
FMA gains by sub-categories (upper-extremity, wrist, hand, coordination). Individual data and average data at post-intervention and follow-up. Larger improvements were observed in the upper-extremity (UE) and wrist categories.

When analyzed by sub-categories, changes in FMA did not happen in the hand category only, and larger improvements were observed in the upper-extremity and wrist categories, Figure 5.

### 3.5. Overall improvement of secondary outcomes after the intervention

In addition to the primary outcome FMA-UE, we were able to observe a general improvement for the secondary outcomes for the whole pool of participants (*n* = 10) over the 4 weeks of robotic intervention and at the follow-up, see Figure 4. Specifically, we observed significant changes for MAL-AOU and COPM with respect to baseline values (*p* < .05; Wilcoxon signed-rank test), with scores above the MCID (likewise for GWMFT, despite not being significant from a statistical point of view). Only grip force slightly reduced at follow-up. Please refer to Supplementary Table S1 for all absolute values.

### 3.6. Automatic assessments reflect clinical assessment improvement

**Figure 6.**
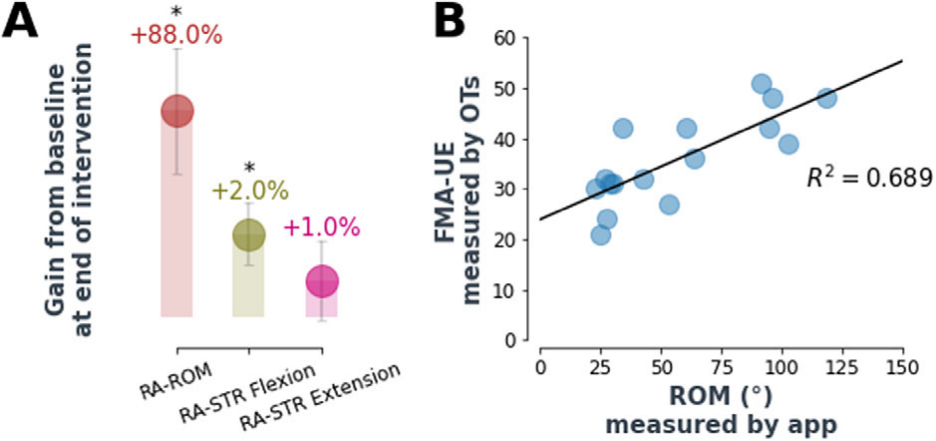
Automatic assessment performance. (a) Robot-assessed max ROM improved by 88% at the end of the robot-assisted therapy, while robot-assessed strength improved respectively by 2% (flexion) and 1% (extension). Values are average percentage improvements and standard errors, for the whole population, with respect to baseline values. Interestingly, the flexion RA-STR at the end of the therapy is in line with the value of grip force as measured by the hand dynamometer (2%). RA-STR is not a direct measure of force but it is inferred from max ROM with inflated actuators (refer to methods for more information). RA-ROM and RA-STR data on one individual were not collected due to a technical issue (*n* = 9). * asterisk indicates statistical significance of the absolute values after Wilcoxon signed-rank test (*p* < .05). (b) RA-ROM correlated with the FMA-UE assessed by an occupational therapist (*R* ^2^ = 0.69). Data points refer to both pre- and post-intervention FMA/ROM assessments (*n* = 9).

Robotic assessments of maximum ROM (RA-ROM) and strength (RA-STR) (see Methods for descriptions) were found to improve (except for RA-STR extension, *p* < .05; Wilcoxon signed-rank test) at the end of the intervention as shown in Figure 6. Looking at the average values of these, RA-ROM improved by 88% after 4 weeks of intervention and flexion RA-STR by 2%. Interestingly, the flexion RA-STR at the end of the therapy is in line with the value of grip force as measured by the hand dynamometer (2%). Additionally, the RA-ROM correlated with the FMA-UE assessed by an occupational therapist (*R* ^2^ = 0.69). RA-ROM and RA-STR data on one individual were not collected due to a technical issue (*n* = 9).

## 4. Discussion

In this work, we have presented a feasibility study on a home-based soft robotic telerehabilitation system for hand rehabilitation post-stroke, including a self-donnable inflatable soft robotic glove and a custom therapy software interface. Two promising outcomes of this intervention were that (i) participants used the system completely unsupervised, and (ii) despite being moderately to severely impaired and in their chronic stage of recovery, performed exercises both at high intensity – rate of over 200 movements in 20 min of exercise on average – and high dosage – 25 sessions over the 28 days of intervention. This is an order of magnitude higher than standard care found in therapy clinics (Lang et al., 2009), and such a level of intensity is strongly believed to be critical for improved recovery (Winstein, 2016).

Previous studies with rehabilitation robots at home did not report data on intensity (i.e., number of movements per hour or per session). Regarding dosage (i.e., hours spent performing robot-assisted therapy), participants used the system 2 hr per week on average, similar to previous works (Nijenhuis et al., 2015, 2016; Radder et al., 2019) (all studies with no strict weekly dosage, as in our protocol), and below (Wolf et al., 2015; Bernocchi et al., 2018), both around 4 h/week due to a stricter increased use guideline. However, apart from Bernocchi et al., (2018), they all reported larger variation between individuals (at least one participant using the robot less than 20 min per week).

The fact that the majority of the participants lived alone, with no possibility to be assisted by a caregiver, highlights the potential for translating the technology and enabling remote physical rehabilitation for this population. Such independent use was not possible in some of the previous studies performed with a rehabilitation robot at home (Nijenhuis et al., 2015, 2016; Bernocchi et al., 2018). In Radder et al. (2019) participants did use the robot independently but were less impaired at baseline. In Wolf et al. (2015), while participants had a similar level of impairment at baseline, high caregiver participation was described even though it is conceivable the device could have been used independently. Importantly, no pain or discomfort issues were reported by the participants at any stage of the intervention and all participants completed the 4-week therapy and the follow-up assessment.

Similarly to previous works, adherence to the program was supported via remote monitoring by the clinical team, who did not directly supervise training sessions. In this study, participants were encouraged to complete a 45-min session (including setup time, donning, and doffing) every time they used the robot but autonomously decided the duration of the intervention to perform. Interestingly, participant engagement – in terms of both dosage and intensity – was not affected by independent use and did not decrease with time, despite virtual check-ins with an OT reducing over the course of the 4 weeks (4 visits to 2 per week). This trend was also reported by Nijenhuis et al. (2015), and it is thus a confirmation of the effectiveness of combining telerehabilitation with robotics and engaging software interfaces.

From a clinical standpoint, all participants reported small but significant improvements in hand motor and functional activity as measured by the primary and secondary outcomes of the study. Importantly, the observed gains are in line with previous literature results involving a chronic (Kim et al., 2019; Cheng et al., 2020) and sub-acute (Milia et al., 2019) stroke population, despite these studies being performed supervised in-clinic and, for Milia et al. (2019) and Cheng et al. (2020), in combination with standard care. Moreover, when compared to previous home-based robotic rehabilitation studies, we observed similar improvements in the primary outcomes while providing shorter therapy duration (a total average of 8 hr versus 28 hr in Bernocchi et al., (2018) and 12 hr in Nijenhuis et al. (2015). In Wolf et al. (2015) and Radder et al. (2019) clinical assessment improvements were higher, but possibly as a consequence of the enrollment of different testing populations (a less impaired population in Radder et al., 2019 where only 19% of participants were stroke survivors, and sub-acute individuals in Wolf et al., 2015), and longer duration interventions (a total of 15 h for Radder et al., 2019, and 36 h for Wolf et al., 2015). Additionally, in Wolf et al. (2015) participants performed a self-administrated home exercise program (including upper-limb exercises of ROM, weight-bearing, exercises with cane, etc.) in addition to the robot-assisted therapy, which likely contributed to gains.

Enrolling a more severely impaired chronic population generally results in lower improvement compared to studies enrolling mildly impaired or subacute/acute individuals (Proietti et al., 2022). The difference may be due to a lack of spontaneous recovery in the chronic population as per acute and sub-acute individuals (Demain et al., 2006), as well as a lack of engagement given the poor visible improvements for those with long-standing impairments. The highly impaired population in this study (five scored below 32 points in FMA-UE at baseline) is representative of a large group of patients – 50% of stroke survivors are chronically disabled with severe impairment, spasticity, and hand tightness (Donkor, 2018) – who require physical assistance to combat severe spasticity and tone. The promising results from this study motivate the design and development of rehabilitation robotics programs at home that can include members of this large community.

Interestingly, we observed improvements in FMA sub-categories not strictly related to the hand performance (upper-extremity and wrist portions). These improvements not only sustained but increased at follow-up. According to literature (Wilkins et al., 2017), individuals with more severe impairments may still benefit from activity-based therapies (such as grasp and release training, as in our glove system protocol), and receive some amount of impairment level improvements throughout the upper limb, due to cortical reorganization as a response to the intervention. Despite the glove mechanically opening and closing the hand specifically, the participants were encouraged to do full arm movements such as targeted reaching before, during and after those treatments, which is possibly the reason why we observed these FMA improvements. A limitation of this study was that we did not monitor participants while not performing the therapy and after the 4 weeks of intervention. This limits our understanding of some observed trends (e.g., those at follow-up) and what different routines this robot-led intervention may have fostered in the participants, and future investigation will be needed to delve into this more thoroughly.

Despite its critical role in hand rehabilitation (Bae et al., 2015), we were only able to observe small improvement in flexion strength at the end of the intervention and this needs further investigation. However, this finding is in line with what was shown previously by other home-based robotic rehabilitation studies (e.g., Nijenhuis et al., 2015). We did observe a significant improvement in the range of movement of the impaired hand, but it is possible that longer duration training or more focused resistance training would be required to increase grip strength. This finding is supported by the results of Radder et al. (2019), who found a sub-group performing specific resistance training did improve grip strength, while a sub-group of participants performing assisted ADLs did not. While not implemented in the current system, inclusion of specific resistance training exercises enabled by the soft robotic glove and guided by the software interface is possible. While flexion strength partially improved, we were not able to observe improvement in extension strength. However, a unique aspect of our approach is that we can assess finger extension strength by leveraging resistance delivered by the soft robotic glove and its integrated sensor. This movement (finger extension) is important for opening of the hand and for performing ADLs but is not measured in current clinical practice or studies as a hand dynamometer only measures flexion strength.

From a technological standpoint, the robotic system used in this study is one of the few examples in literature (Wolf et al., 2015; Nijenhuis et al., 2016) of home-based connected robotic telerehabilitation device which allows for real-time remote data monitoring by the research team, including occupational therapists, and as final session report to the participants. This approach is an example of a design feature that was heavily influenced by the feedback collected from both stroke survivors and therapists during the focus groups and in responses to surveys. The cloud-connection allowed our therapists to access at any time the data from the sensorized glove (e.g., finger ROM, actuator pressure), app-measured assessments, time of use, number of repetitions, and so forth. In this study, this useful information was utilized to encourage adherence to the program. Participants received an automatic in-app report at the end of every session, that may have supported their strong engagement. Noticeably, providing data directly to the patients has the potential to enhance a behavioral change, as the participants can directly observe changes in their scores thought to be caused by their adherence to the rehabilitation protocol.

The ability to measure quantitative data from the user performance allow for inter-session longitudinal analysis of the patient data, thus opening up the possibility for remote therapeutic monitoring (RTM). RTM is an emerging aspect of the healthcare landscape, showing promising results toward improving rehabilitative results (Halloran et al., 2019; Postolache et al., 2021). By digitally sharing progress data, the “*silo effect*” of today’s medicine landscape, in which time and valuable information are lost between the many locations and providers who see the same client, could be reduced (Meneses and Caseiro, 2018). Critically, we do not envision this technology replacing the role of an occupational or physical therapist, but rather believe RTM, through a technology such as our soft robot, can augment their ability to offer better care and effectively treat a larger number of patients. In a commercial application, RTM also offers additional revenue streams by clinicians billing third-party payers for reviewing and interacting with user data including program adherence and response.

From a usability point of view, all participants enjoyed using the system, as indicated by the SUS, and wanted to continue use beyond the 4-week intervention. They reported finding the system more engaging than conventional home therapy strategies such as completing a recommended list of rote exercises (Donoso Brown et al., 2015). The average SUS score (77), was in line with previous assistive/rehabilitative published technologies used unsupervised at home (69–73 in Radder et al., 2019 and 69 in Nijenhuis et al., 2015) and indicates high probability for acceptance in the field (Bangor et al., 2009). These promising results are likely the positive consequences of the user-centric design of the robot and of the software interface.

A limitation of the current study concerned the automatic estimation strategies (strength and maximum ROM). We were able to measure maximum ROM (e.g., 80° of hand motion), but not absolute values of ROM (e.g., 30° of extension and 50° of flexion) due to the nature of the sensor and the unsupervised condition of the experiment. Moreover, the bend sensor was incorporated into the index finger only, thus giving a limited overview of the whole hand ROM. Similarly, with the glove inflated to a fixed pressure, we were able to measure changes in maximum ROM for flexion and extension as a proxy for hand strength but the output is not directly related to a quantifiable measurement of force. However, despite these limitations, having the ability to measure relative changes in hand ROM and strength over time remotely seems feasible. The current version of the glove actuates all fingers simultaneously, while most of the interviewed OTs and PTs underlined the importance of individual actuation of the fingers, which can be explored in future design iterations. Moreover, during active games the control strategy allowed the user to actively trigger the inflation of the glove, either in flexion or extension, to a set pressure. An advanced control allowing customized assistance modulation may be needed in future to even further increase engagement by the participants and perhaps the outcomes of the therapy. Being an exploratory pilot study, the size of the testing group was small and future investigation will be needed to confirm results on a larger cohort. Moreover, the lack of a control group performing standard care at home, limited the possibility to compare results and judge the effectiveness of the presented system. This will also be targeted in future investigations. Finally, the OTs who assessed the participants pre/post intervention were not blinded to the study protocol, thus independent evaluation should be considered in the future.

This research demonstrates a promising preliminary step toward an efficacious home-based robotic rehabilitation technology for people in need of rehabilitation and motor assistance, such as individuals with chronic stroke and severe hand impairment. The outcomes presented in this study highlight the potential for a shift for physical rehabilitation from the clinic to the home, greatly reducing the burden on patients (i.e., travel, scheduling), while still allowing monitoring of the intervention through quantitative data collected by the robot, and thus customization of therapy on the specific patient’s needs.

**Nomenclature**
ARAT: Action Research Arm Test
BBT: Box and Block Test
COPM: Canadian Occupational Performance Measure
FMA-UE: Fugl-Meyer Assessment-Upper Extremity
GWMFT: Graded Wolf Motor Function Test (Functional Ability FAS, Performance Time TIME)
JTHFT: Jebsen-Taylor Hand Function Test
MAL: Motor Activity Log
MAS: Modified Ashworth Scale

## Supporting information

Proietti et al. supplementary material Proietti et al. supplementary material

## Data Availability

The datasets generated during and/or analyzed during the current study are available from the corresponding author upon reasonable request.

## Supplementary material

The supplementary material for this article can be found at https://doi.org/10.1017/wtc.2023.26.
