# Supplementary material for: Combining soft robotics and telerehabilitation for improving motor function after stroke
Source: Wearable Technol. 2024 Jan 26;5:e1. doi: 10.1017/wtc.2023.26 (PMC10952055; doi:10.1017/wtc.2023.26)
Supplement: Proietti et al. supplementary material [file S2631717623000269sup001.docx]

**Combining soft robotics and telerehabilitation for improving motor function after stroke**

Proietti T.^1^*, Nuckols K.^1^*, Grupper J.^1^, Schwarz D.^1^, Inirio B.^1^, Porazinski K.^2^, Wagner D.^1^, Cole T.^1^, Glover C.^1^, Mendelowitz S.^1^, Herman M.^1^, Breen J.^2^, Lin D.^3,4^, Walsh C.^1†^

# **SUPPLEMENTARY MATERIAL**


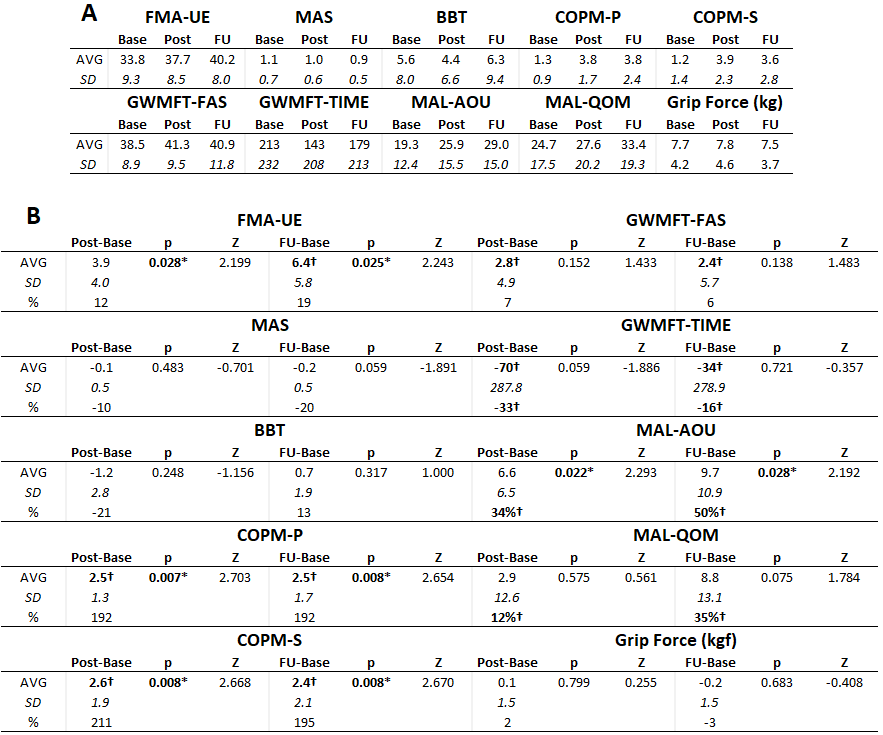


**Table 1: Clinical assessments at baseline, post-intervention (after 4 weeks of home therapy), and follow up (2 weeks after post-intervention, 6 weeks from baseline).** A) Average and standard deviation, absolute values. B) Average, standard deviation, and percentage, values relative to baseline. * asterisk indicates statistical significance after Wilcoxon signed-rank test (p-value < 0.05). ✝ dagger indicates results over the MCID (FMA = 5.25 [Page2012], MAS = 0.48 [Chen2019], BBT = 5.5 [Chen2009], COPM = 2 [Tuntland2016], GWMFT-FAS = 1 / 17% [Lang2008], GWMFT-TIME = 19s / 16% [Lang2008], MAL = 10% [Hung2022], Grip Force = 5kg [Lang2008]).
